# Can salivary microbiome become a biodetector for type-2 diabetes? Opinion for future implications and strategies

**DOI:** 10.3389/fnut.2023.1113591

**Published:** 2023-01-19

**Authors:** Hardinsyah Hardinsyah, Fahrul Nurkolis, Rudy Kurniawan, William Ben Gunawan, Piko Satria Augusta, Astuti Setyawardani, Rafiv Fasya Agustianto, Msy Firyal Nadya Al Mahira, Ghevira Naila Praditya, Deogifta Graciani Lailossa, Dewangga Yudisthira, Salsabila Farradisya, Hero Barazani

**Affiliations:** ^1^Division of Applied Nutrition, Faculty of Human Ecology, Department of Community Nutrition, IPB University, Bogor, Indonesia; ^2^Department of Biological Sciences, Faculty of Sciences and Technology, State Islamic University of Sunan Kalijaga (UIN Sunan Kalijaga Yogyakarta), Yogyakarta, Indonesia; ^3^Alumnus of Department of Internal Medicine, Faculty of Medicine, University of Indonesia–Cipto Mangunkusumo Hospital, Jakarta, Indonesia; ^4^Alumnus of Department of Nutrition Science, Faculty of Medicine, Diponegoro University, Semarang, Indonesia; ^5^Medical Study Programme, Faculty of Medicine, Brawijaya University, Malang, Indonesia; ^6^Medical Student of Faculty of Medicine, University of Jember–Soebandi Regional Hospital, Jember, Indonesia; ^7^Medical Student of Faculty of Medicine, Universitas Airlangga, Surabaya, Indonesia

**Keywords:** oral microbiome, biodetector, diabetes, T2DM, salivary bacterial

## 1. Introduction

Microbiome has become a topic that is developing rapidly in the health sector for the past 2 decades (1). Previous research has found that the microbiome was associated with a variety of diseases, such as metabolic, gastrointestinal, and immunity disorders (2). The microbiome is a collection of genomes or genetic materials from all microorganisms, symbiotics, and pathogens that live in vertebrates such as bacteria, viruses, archaea, fungi, and small protists (1, 3). About 100 trillion microbiomes called the gut microbiome to reside in the digestive tract (4). The gut microbiome plays a role in nutrient and drug metabolism, immune modulation, and maintenance of gut integrity (3).

In addition to the gut, the second highest number of microbiomes is located in the mouth and called oral microbiome or salivary bacteriome, considering that there is a link between the two both physically and chemically (5, 6). An oral cavity is an ideal place for the survival of the oral microbiome since it has an average temperature of 37°C and saliva with a stable pH of 6.5–7, causing the bacteria to hydrate transporting the nutrients to microorganisms (7). The oral cavity is the first part of the gastrointestinal tract and the place where it meets with food, exogenous microbes, and allergens before it gets into the gastrointestinal tract. The presence of direct exposure in the absence of fibrous epithelium makes the oral cavity susceptible to infection (8). Those conditions highlight the important role of the microbiome in maintaining ecological balance and preventing oral cavities (9). One of the mechanisms is resistance to the colonization of pathogens by defeating pathogenic species and lowering the chance of integration by exogenous pathogens. Some microbiomes are also able to fight acids produced by caries-causing bacteria by increasing the pH of saliva through the production of alkaline metabolic byproducts (8, 10). Disorder in this system may cause dysbiosis triggered by various factors such as diet, inflammatory response, systemic disorders, and alcohol which will induce oral diseases (5, 8).

Recently, it has been found that the human microbiome has an important role in the occurrence and development of diabetes mellitus (11). Several studies have shown that type 2 diabetes mellitus (T2DM) is associated with changes in the diversity and number of bacteria in supragingival plaques, and oral microbiota changes have also been found in various glycemic states (12, 13). The phylum *Actinobacteria* was found to have decreased in the T2DM group compared to the control group, and the increase was associated with a reduced risk of T2DM (11, 14). The phylum *Actinobacteria* is also associated with the prevalence of obesity, suggesting that the oral microbiome may have an important role in the etiology of diabetes (14). The number of microbiotas of the genus *Prevotella* was decreased in the T2DM group, and *Prevotella spp.* was reduced in high-glucose salivary conditions (11, 12). Bacteria of the genus *Rothia* experienced a decrease in T2DM and potential pre-diabetic conditions (11, 15). Bacteria in the phylum *Firmicutes*, one of which is the *Streptococcus*, was significantly increased in the T2DM and pre-diabetic groups compared to the non-diabetic group (11, 13, 16).

To the best of our knowledge, there has been no review study or opinion article related to the use of oral or salivary microbiome as a biodetector of T2DM disease. Therefore, the main purpose of this critical opinion is to summarize the findings regarding the oral microbiome as a biodetector of T2DM and explain its opportunities, implications, and strategies for future use.

## 2. Oral microbiome in general

The human mouth is inhabited by various microorganisms such as bacteria, viruses, fungi, and protozoa called oral microbiota. The diversity of the human oral microbiota is one of the effects of accelerated regeneration and non-keratinized epithelium types found in the oral cavity (17, 18) since these factors increase the process of molecular absorption, implying that the microbiota is more likely to reach other organs and has a broad metabolic effect. In addition, the diversity of the oral microbiota can also be caused by the high intensity of contact with the external world *via* air and food (18, 19). Assuming the health-related consequences of the composition of the oral microbiota, the oral cavity would be an ideal place to analyze biomarkers since the samples will be easier to obtain than other organs (20). To date, 445 types of oral microbiota have been recognized in literature, which is ranked second after the intestine and 57% of them have been named and perfectly cultured (21, 22).

Due to its diverse and easily detectable natures, oral microbiota can be used as a biomarker of some diseases in humans (18). In diseases related to children’s mouths, some fungal species such as *Candida dubliniensis* and *Candida tropicalis* in saliva are indicators that children are at risk of dental caries (23). Changes in the oral microbiota to acidogenic and acidic cariogenic bacteria can indicate tooth decay and enamel demineralization in children (24). In adults, the oral microbiota can be an indicator of the incidence of oral cancer since the bacterial composition changes due to a cluster of factors that cause oral cancer, including alcohol and cigarettes. The byproducts of oral bacteria can induce genetic changes in mucosal epithelial cells that are predictors of Squamous cell carcinoma in the mouth (25). Further findings found that the use of oral microbiota was able to detect the presence of oropharyngeal cancer and malignancy in the human gastrointestinal tract (7, 26).

In addition, oral microbiota can also be an important biomarker of systemic diseases in other organs (27). Oral microbiota has also been detected in the lungs in cystic fibrosis patients and can cause various soft tissue infections in the event of wound bites (28, 29). *Selenomonas* (*S. artemidis* and *S. infelix*) can be used as important biomarkers of lung infections associated with acute respiratory distress syndrome (ARDS) (30). The oral microbiota has also long been known as a reservoir of infections in various parts of the body. The appearance of abscesses in the brain can also be detected with the discovery of an increase in *Porphyromonas gingivalis* which is an etiological agent in periodontitis (31). The study with 228 subjects diagnosed with metabolic syndrome disease in Korea also showed significant differences where the group with metabolic syndrome had slightly more *Firmicutes* (37.9%) and slightly fewer *Proteobacteria* (29.2%) than the healthy group (21). In other studies, several dominant oral microbiotas were found such as *Fusobacterium nucleatum*, *Veillonella*, *Streptococcus anginosus*, *Streptococcus oralis*, and *Actinomyces meyeri* in pyogenic infectious diseases of the brain and spinal cord (30, 32). Looking at these findings drew attention to the question “Can the oral profile of the microbiota be used as a diagnostic biomarker or biodetector of T2DM?” Following the purpose of this opinion article, the author tries to summarize the findings regarding the oral microbiome as a biodetector of T2DM.

## 3. Is the oral microbiome effective in detecting T2DM?

### 3.1. *In vivo* or preclinical trial studies

The decline of the body’s immune system in people with diabetes mellitus occurs due to polymorphonuclear cell dysfunction (PMN) and various other inflammatory cytokines, which causes a shift in the normal flora of the oral mucosa and triggers the growth and development of various pathogenic germs (33). Xiao et al. conducted *in vivo* experiments on rats treated to be hyperglycemic. This study found elevated levels of *Enterobacteriaceae*, *Aerococcus*, *Enterococcus*, and *Staphylococcus* which are often associated with T2DM (34). The human and mouse microbiomes have identical compositions at the phylum level, including *Firmicutes* and *Bacteroidetes*, followed by *Actinobacteria* and *Proteobacteria* In addition, at the genus level, *Lactobacillus* is the dominant genus at 8 weeks of age and remains dominant during the development of T2DM (35, 36).

### 3.2. Is there sufficient clinical evidence or clinical trials?

Table 1 showed that some of these studies showed contrasting findings. Almeida-Santos et al. and Lee et al. found no difference in oral microbiome profiles between diabetic and non-diabetic groups (37, 38). However, it can be concluded that several oral microbiomes are quite often found in T2DM, including the *Firmicutes* and *Bacteroidetes* phylum, followed by *Actinobacteria* and *Proteobacteria*. Based on *in vivo* and clinical studies, the mechanism of identification and the correlation between the oral microbiome and the occurrence of T2DM still need to be further researched, especially considering the possible differences between ethnicities or populations (43). It is realized, the insignificant of studies in Table 1 were likely underpowered because perhaps the number of samples was relatively small (<50 patients). Future clinical trial studies or RCTs should be conducted with more power based on sample size, may more than fifty patients or subject.

**TABLE 1:** List of clinical evidence regarding the effectivity of oral microbiome in detecting type 2 diabetes mellitus (T2DM).

No	Studies	Outcomes	References
1	Characterization of the type of oral microbiome in T2DM patients (RNA sequencing method, *n* = 46); BMI or Body Mass Index (kg/m2) defined as normal weight, preobese, obese, and the obese category had a higher Shannon index for the abundance of amplicon sequence variants (ASVs), followed by the preobese category.	∙ Based on phylum, Oral microbiomes that are detected in T2DM: *Firmicutes* (45%) ↑, *Bacteroidetes* (22%)↑, *Proteobacteria* (16%)↑, *Actinobacteria* (9%)↑, and *Fusobacteria* (6%)↑. ∙ Based on the genus, the oral microbiome is dominated by *Streptococcus* (29%)↑, *Prevotella* (14%)↑, and *Neisseria* (5%)↑. ∙ There were no significant differences in the oral microbiome profiles in diabetic and non-diabetic patients. ∙ There was no significant difference in alpha and beta diversity levels in diabetic and non-diabetic groups.	Almeida-Santos et al. (37)
2	Characterization of the type of oral microbiome in T2DM patients (DNA sequencing method, *n* = 26); BMI 25.0 ± 1.3 (Diabetes group; *n* = 10) and 24.6 ± 2.2 (Non-diabetes group; *n* = 13).	∙ Based on the phylum, the oral microbiome is dominated by *Firmicutes*↑, while *Streptococcus*↑ dominates the genus level. ∙ There was no significant difference in alpha and beta diversity levels in diabetic and non-diabetic groups.	Lee et al. (38)
3	Characterization of the type of oral microbiome in T2DM patients (DNA sequencing method, *n* = 24); BMI in this study was not reported.	∙ Diabetes mellitus causes environmental changes and a shift in microbial homeostasis of the oral mucosa. ∙ Oral microbiomes that are detected in DMT2 patients include: *Bacillus mojavensis*↑, *Enterobacter cloacae*, *Proteus mirabilis*↑, *Staphylococcus epidermidis*↑, *Staphylococcus hominis*↑, *Staphylococcus pasteuri*↑, *Streptococcus mutans*↑, and *Streptococcus pasteurianus*↑.	Ali et al. (39)
4	Changes in oral microbiome in periodontitis in T2DM and non-diabetic patients (RCT study, *n* = 133); BMI was observed for each group.	∙ The proportion of *Prevotella copri*↑, *Alloprevotella rava*↑, and *Ralstonia pickettii*↑ increased in patients with periodontitis in both T2DM and non-diabetic groups. ∙ Moreover, the oral microbiome will decrease once glucose levels in the patient are controlled.	Sun et al. (40)
5	Characterization of the type of oral microbiome in T2DM patients (DNA sequencing method, *n* = 128); BMI was observed for each group, 24.9 ± 5.7 in Normoglycemic (*n* = 32) and >30.0 in Pre-DM and DM groups.	∙ *Fusobacteria* and *Actinobacteria* are significantly more abundant (↑) than *Proteobacteria* (↓) in diabetic subjects.	Matsha et al. (13)
6	Characterization of salivary microbiota in elderly patients with T2DM (RNA metagenomic analysis, *n* = 84); As a matched case–control study and BMI in this study were not reported.	∙ The phylum *Firmicutes*↑ was abundant in patients with T2DM, whereas the phylum *Bacteroidetes*↑ was abundant in controls.	Omori et al. (41)
7	Characterization of oral microbiome profile of Chinese patients with T2DM (RNA sequencing, *n* = 442); 20.1 ± 1.2 in Control/Health groups and 27.1 ± 0.8 in T2DM	∙ The *Firmicutes*/*Bacteroidetes*↑ ratio increased in T2DM. ∙ T2DM patients presented significantly higher numbers of *Neisseria*↑, *Streptococcus*↑, *Haemophilus*↑, and *Pseudomonas*↑, and lower numbers of *Acinetobacteria*↓ compared with healthy controls.	Chen et al. (42)

↑ Indicates an increase in number or abundance, and ↓ indicates a decrease in the number of oral bacteria.

## 4. Discussion with future implications and strategies

**FIGURE 1:**
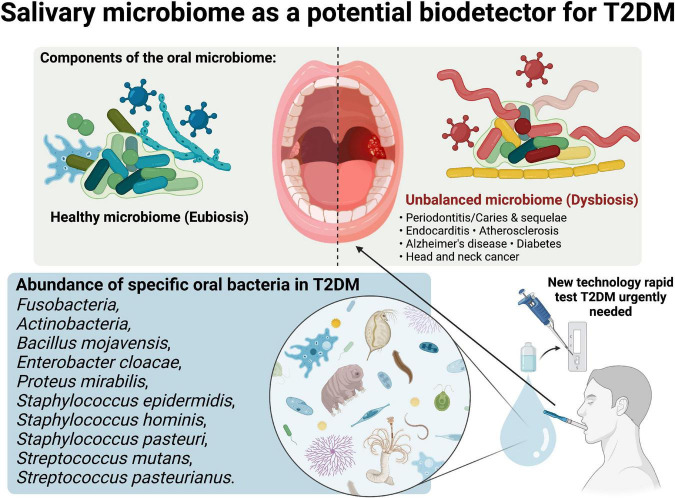
Salivary microbiome as a potential biodetector for type 2 diabetes mellitus (T2DM). Created with BioRender.com premium license by Fahrul Nurkolis.

Previous studies has highlighted the interesting bidirectional relationship between the salivary microbiome and T2DM (44). The abundance of *Firmicutes* and increased ratio of *Firmicutes*/*Bacteroidetes* were found in most studies. It has been suggested that the *Firmicutes* were more efficient than the *Bacteroidetes* at obtaining energy from food, resulting in more effective absorption of calories and the ensuing weight gain (45). On the other hand, dietary intake and obesity were correlated with the incidence of T2DM (46). A lower level of *Bacteroidetes* has also been linked with the incidence of inflammatory disease (47), while inflammation also initiates the pathophysiology of T2DM (48). However, even this fundamental theory was found to be contradicted as *Firmicutes* was argued to be health-promoting bacteria through the synthesis of butyrate (49). Lee et al., Chen et al. and Ali et al. also mentioned that *Streptococcus* was one of the abundant bacteria in the oral microbiome of T2DM subjects (Figure 1) (38, 39, 42). Lower *Prevotella* and higher *Streptococcus* may be explained by low salivary pH that is caused by prolonged elevated blood glucose (12). However, a clinical research report stated that salivary microbiome profiles were prone to change, as they might be influenced by different stages of oral diseases (13). This was confirmed by the study of Sun et al. (40) as the changes of bacteria between diabetic and non-diabetic groups were similar in the presence of periodontitis.

Even though oral microbial profiles exhibited wide metabolic implications that were similar but at a lower level compared to gut microbiome (50), they may contribute an important role as biodetector of diseases, especially T2DM. Learning from the studies reviewed in this opinion, it can be concluded that randomized controlled trials (RCTs) are needed to consolidate the evidence regarding the link between oral microbiome and T2DM. These studies presented us with insights regarding related bacteria that can be targeted during upcoming RCTs, as these bacteria have been studied for their correlation with impaired metabolical conditions such as T2DM. Profiling the salivary microbiome may become a feasible rapid test in detecting T2DM (Figure 1), considering the fact that bio samples of the oral cavity will be easier to procure compared to gut (fecal) microbiota for future use, compared to detection using blood which some people are afraid of needles.

## Author contributions

HH and FN: conception and design of opinion studies. FN: figure visualization and BioRender license holder. All authors wrote, edited, and revised the manuscript, and approved the final version of the submitted manuscript.
